# Supplementary material for: A virus-encoded type I interferon decoy receptor enables evasion of host immunity through cell-surface binding
Source: Nat Commun. 2018 Dec 21;9:5440. doi: 10.1038/s41467-018-07772-z (PMC6303335; doi:10.1038/s41467-018-07772-z)
Supplement: Supplementary file 1 — Supplementary Information [file 41467_2018_7772_MOESM1_ESM.pdf]

Supplementary Information

Cell surface binding activity is required for efficient evasion of host immunity by a virus-encoded type I IFN decoy receptor.

Hernaez et al.

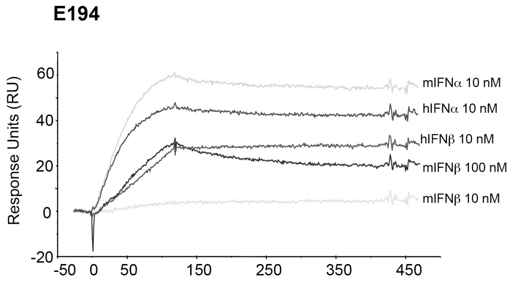

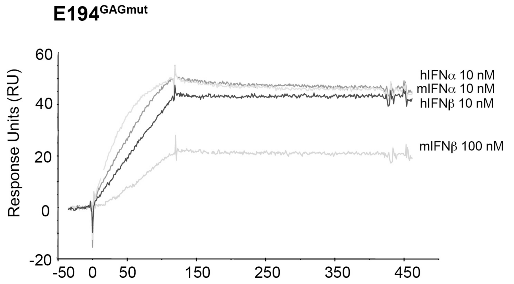

Supplementary Figure 1. E194 and E194^{GAGmut} do not efficiently bind to mIFNβ. SPR sensorgrams obtained for the determination of the interaction of E194 and E194^{GAGmut} recombinant proteins with mouse IFNβ. E194 or E194^{GAGmut} were immobilized in a SPR Biacore SA sensor chip and binding and dissociation of indicated concentrations of mIFNα, mIFNβ, hIFNα and hIFNβ at 30 μl/min were recorded.

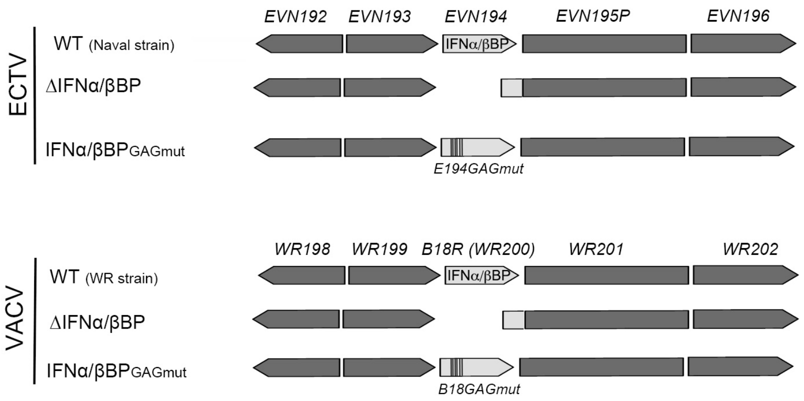

Supplementary Figure 2. Schematic representation of the genomic organization of the recombinant poxviruses used in this work.

a

Dose (pfu/animal)	ECTV WT (Naval)	ECTV ΔIFNα/βBP	ECTV IFNα/βBP^{GAGmut}
1	2/5	ND	ND
10	0/5	ND	5/5
10^2	ND	ND	5/5
10^3	ND	ND	5/5
10^4	ND	ND	5/5
10^5	ND	5/5	5/5
10^6	ND	5/5	4/5

b

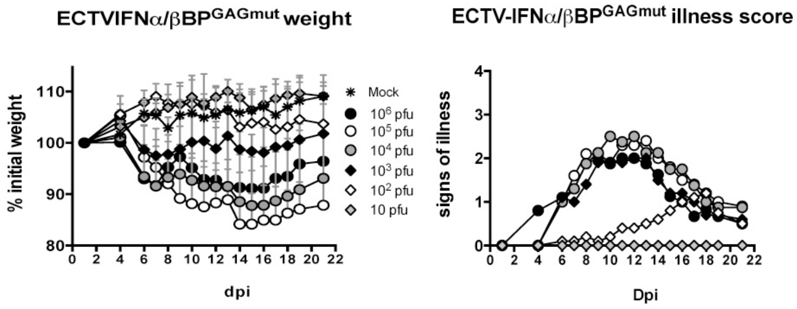

Supplementary Figure 3. Mousepox after infection with increasing doses of ECTV. Balb/c mice were s.c. inoculated in the footpad with increasing doses of the ECTV IFNα/βBP mutants. Survival rates (A) and weight loss and mousepox clinical signs (B) were evaluated. Weight loss is expressed as mean +/- SD of the five animal weights compared to their original weight at the day of inoculation.

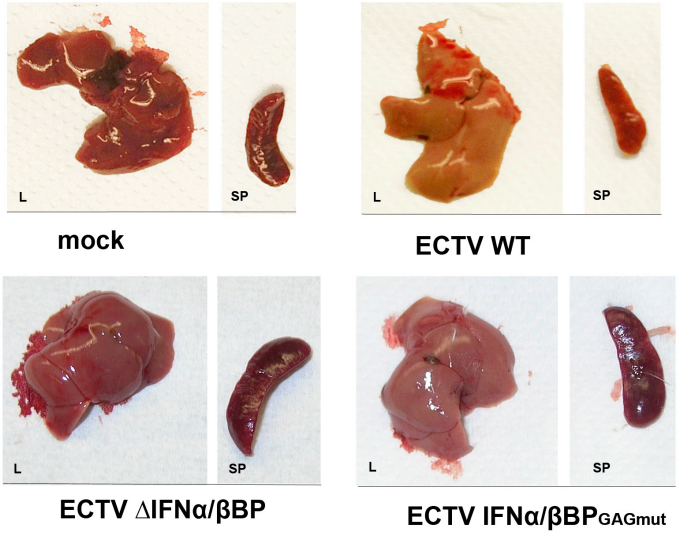

Supplementary Figure 4. Spleen (SP) and liver (L) from Balb/c mice at 7 dpi after s.c. inoculation in the footpad with 10^3 pfu of the indicated ECTV. Macroscopic morphological changes observed after wild type ECTV infection consisted of a reduction in spleen size and pale in colour with extensive necrotic signs in both organs, as compared to mock-infected mice. On the contrary, spleens of animals infected with ECTVΔIFNα/βBP were enlarged and both organs presented a normal colour. Organs from ECTVIFNα/βBP^{GAGmut} infected animals exhibited an intermediate appearance: spleens were enlarged, red coloured with some evident signs of necrosis (white spots), while some discolouration and white necrotic spots were observed in the livers.

	**WT *vs* ΔIFNα/βBP**		**WT *vs* IFNα/βBP^{GAGmut}**	
	upregulated DSEGs	ISGs (%)	upregulated DSEGs	ISGs (%)
**Lymph nodes**	67	58 (86,5%)	214	136 (63,5 %)
**Spleen**	880	273 (31 %)	444	177 (39.8 %)
**Lung**	14	14 (100 %)	25	25 (100 %)

Supplementary Table 1. Proportion of ISGs differentially expressed after i.n. infection with the VACV IFNα/βBP mutants.
