# Supplementary material for: A virus-encoded type I interferon decoy receptor enables evasion of host immunity through cell-surface binding
Source: Nat Commun. 2018 Dec 21;9:5440. doi: 10.1038/s41467-018-07772-z (PMC6303335; doi:10.1038/s41467-018-07772-z)
Supplement: Supplementary file 3 — Reporting Summary [file 41467_2018_7772_MOESM3_ESM.pdf]

nature research

Corresponding author(s): *Double-blind peer review submissions: write DBPR and your manuscript number here instead of author names.*

Reporting Summary

Nature Research wishes to improve the reproducibility of the work that we publish. This form provides structure for consistency and transparency in reporting. For further information on Nature Research policies, see Authors & Referees and the Editorial Policy Checklist.

Statistical parameters

When statistical analyses are reported, confirm that the following items are present in the relevant location (e.g. figure legend, table legend, main text, or Methods section).

n/a	Confirmed	
☐	☒	The exact sample size (n) for each experimental group/condition, given as a discrete number and unit of measurement
☐	☒	An indication of whether measurements were taken from distinct samples or whether the same sample was measured repeatedly
☐	☒	The statistical test(s) used AND whether they are one- or two-sided<br>*Only common tests should be described solely by name; describe more complex techniques in the Methods section.*
☐	☒	A description of all covariates tested
☒	☐	A description of any assumptions or corrections, such as tests of normality and adjustment for multiple comparisons
☐	☒	A full description of the statistics including central tendency (e.g. means) or other basic estimates (e.g. regression coefficient) AND variation (e.g. standard deviation) or associated estimates of uncertainty (e.g. confidence intervals)
☐	☒	For null hypothesis testing, the test statistic (e.g. F, t, r) with confidence intervals, effect sizes, degrees of freedom and P value noted<br>*Give P values as exact values whenever suitable.*
☒	☐	For Bayesian analysis, information on the choice of priors and Markov chain Monte Carlo settings
☒	☐	For hierarchical and complex designs, identification of the appropriate level for tests and full reporting of outcomes
☒	☐	Estimates of effect sizes (e.g. Cohen's d, Pearson's r), indicating how they were calculated
☐	☒	Clearly defined error bars<br>*State explicitly what error bars represent (e.g. SD, SE, CI)*

Our web collection on statistics for biologists may be useful.

Software and code

Policy information about availability of computer code

Data collection	no software was used, except (1) SPR analysis where BIAevaluation 3.2 software was used; and (2) CellQuestPro that was used to collect data of flow cytometry
Data analysis	BIAevaluation 3.2 software was used for SPR experiments. Flowjo was used for flow cytometry. Statisticcal analysis was done with GraphPathD Prism software. For RNAseq analysis we used: Tophat v2.0.4 ; Cufflinks v2.1.0 software ; Ingenuity Pathway Analysis (IPA) software ; and R package "Gplots". As described in the Methods section.

For manuscripts utilizing custom algorithms or software that are central to the research but not yet described in published literature, software must be made available to editors/reviewers upon request. We strongly encourage code deposition in a community repository (e.g. GitHub). See the Nature Research guidelines for submitting code & software for further information.

Data

Policy information about availability of data

All manuscripts must include a data availability statement. This statement should provide the following information, where applicable:

- Accession codes, unique identifiers, or web links for publicly available datasets
- A list of figures that have associated raw data
- A description of any restrictions on data availability

The data that support the findings of this study are available from the corresponding author upon reasonable request. The viral genomic sequences reported, together with the fastq files containing the reads from the RNA-seq experiments have been submited to the European Nucleotide Archive under reference number PRJEB26437 (https://www.ebi.ac.uk/ena/data/view/PRJEB26437).

Field-specific reporting

Please select the best fit for your research. If you are not sure, read the appropriate sections before making your selection.

☒ Life sciences ☐ Behavioural & social sciences ☐ Ecological, evolutionary & environmental sciences

For a reference copy of the document with all sections, see nature.com/authors/policies/ReportingSummary-flat.pdf

Life sciences study design

All studies must disclose on these points even when the disclosure is negative.

Sample size	Our past expertise with the infection model shows that 5 mice/group are necessary to reach statistically significant results. For virus titration experiments, flow cytometry, RNAseq ... we know from previous experience that duplicate samples are sufficient.
Data exclusions	No data were excluded.
Replication	All experiments were replicated as indicated in the Results section and Figure legends.
Randomization	mice were randomly distributed in groups as they arrived to our animal house facility.
Blinding	Blinding is not possible in our animal experiments. We work under BSL3 containment facilities for animal experimentation and we need to avoid cross-contamination of virus recombinants with different virulence degree between mouse groups. We inactivate hood and all material used when we change to groups of mice infected with a different recombinant virus. Cross-contamination would mask any result.

Reporting for specific materials, systems and methods

Materials & experimental systems

n/a	Involved in the study	
☐	☒	Unique biological materials
☐	☒	Antibodies
☐	☒	Eukaryotic cell lines
☒	☐	Palaeontology
☐	☒	Animals and other organisms
☒	☐	Human research participants

Methods

n/a	Involved in the study	
☒	☐	ChIP-seq
☐	☒	Flow cytometry
☒	☐	MRI-based neuroimaging

Unique biological materials

Policy information about availability of materials

Obtaining unique materials	All materials are available from authors upon request or from commercial sources.

Antibodies

Antibodies used	monoclonal anti-V5 antibody (Invitrogen) Cat. No. R960-25; anti-mouse IgG-A488 (Molecular Probes) Cat. No. A21202.

Validation | These antibodies are purchased and distributed by the Flow Cytometry Service from Centro de Biologia Molecular Severo Ochoa.

Eukaryotic cell lines

Policy information about cell lines

Cell line source(s)	BSC-1 (ATCC CCL-26), HeLa (ATCC CCL-2), Vero (ATCC CCL-81), mouse L929 cells (ATCC CCL-1) and CHO-K1 cells were kindly provided by Dr. Arenzana-Seisdedos (Institute Pasteur, Paris, France)
Authentication	None of the cell lines were authenticated
Mycoplasma contamination	All cell lines were confirmed to be mycoplasma negative in regular tests.
Commonly misidentified lines (See ICLAC register)	Do not apply

Animals and other organisms

Policy information about studies involving animals; ARRIVE guidelines recommended for reporting animal research

Laboratory animals	mouse, Balb/c, female, 5-6 weeks old.
Wild animals	The study did not involve wild animals.
Field-collected samples	The study did not involve samples collected from the field.

Flow Cytometry

Plots

Confirm that:

- [ ] The axis labels state the marker and fluorochrome used (e.g. CD4-FITC).
- [x] The axis scales are clearly visible. Include numbers along axes only for bottom left plot of group (a 'group' is an analysis of identical markers).
- [x] All plots are contour plots with outliers or pseudocolor plots.
- [ ] A numerical value for number of cells or percentage (with statistics) is provided.

Methodology

Sample preparation	CHO-K1 cells were detached with 4 mM EDTA at 37ºC and harvested in PBS. Cells (3x105 per experimental point) were incubated for 30 min on ice with 250 nM of the indicated viral recombinant proteins. Cells were then extensively washed with FACS buffer (PBS, 0.01% sodium azide and 0.5% bovine serum albumin) and incubated for 30 min at 4ºC with monoclonal anti-V5 antibody (Invitrogen) diluted 1:500 followed by anti-mouse IgG-A488 (Molecular Probes) diluted 1:500 in PBS.
Instrument	FACSCalibur flow cytometer (BD Sciences)
Software	CellQuestPro that was used to collect data of flow cytometry; Flowjo was used for flow cytometry analysis
Cell population abundance	100% of cells analyzed were the same, we analyzed a cell line.
Gating strategy	No gating was required since all cells were the same.

- [ ] Tick this box to confirm that a figure exemplifying the gating strategy is provided in the Supplementary Information.
